# A synapsin Ⅰ cleavage fragment contributes to synaptic dysfunction in Alzheimer's disease

**DOI:** 10.1111/acel.13619

**Published:** 2022-04-20

**Authors:** Lanxia Meng, Li Zou, Min Xiong, Jiehui Chen, Xingyu Zhang, Ting Yu, Yiming Li, Congcong Liu, Guiqin Chen, Zhihao Wang, Keqiang Ye, Zhentao Zhang

**Affiliations:** ^1^ 117921 Department of Neurology Renmin Hospital of Wuhan University Wuhan China; ^2^ 89674 Department of Neurology Zhongnan Hospital of Wuhan University Wuhan China; ^3^ 12239 Department of Pathology and Laboratory Medicine Emory University School of Medicine Atlanta Georgia USA

**Keywords:** Alzheimer's disease, asparagine endopeptidase, synapsin Ⅰ, synaptic dysfunction

## Abstract

Synaptic dysfunction is a key feature of Alzheimer's disease (AD). However, the molecular mechanisms underlying synaptic dysfunction remain unclear. Here, we show that synapsin Ⅰ, one of the most important synaptic proteins, is fragmented by the cysteine proteinase asparagine endopeptidase (AEP). AEP cleaves synapsin at N82 in the brains of AD patients and generates the C‐terminal synapsin Ⅰ (83–705) fragment. This fragment is abnormally distributed in neurons and induces synaptic dysfunction. Overexpression of AEP in the hippocampus of wild‐type mice results in the production of the synapsin Ⅰ (83–705) fragment and induces synaptic dysfunction and cognitive deficits. Moreover, overexpression of the AEP‐generated synapsin Ⅰ (83–705) fragment in the hippocampus of tau P301S transgenic mice and wild‐type mice promotes synaptic dysfunction and cognitive deficits. These findings suggest a novel mechanism of synaptic dysfunction in AD.

## Graphical abstract

Asparagine endopeptidase is activated in an age‐dependent manner in the brain. Active AEP cleaves synapsin I and generates the synapsin I C83 fragment, which affects the recycling of synaptic vesicles and causes synaptic dysfunction and cognitive impairment, promoting the onset of AD.

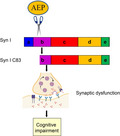

## 1 INTRODUCTION

Alzheimer's disease (AD) is the most common age‐related neurodegenerative disease. The major clinical manifestation of AD is progressive cognitive decline. Pathologically, AD is characterized by synaptic dysfunction and the accumulation of amyloid plaques and neurofibrillary tangles. Synaptic dysfunction is one of the major contributors to the symptoms of AD (Bastrikova et al., 2008; Davies et al., 1987; Morrison & Baxter, 2012; Selkoe, 2002). Moreover, synaptic dysfunction is a very early feature of AD and occurs years before the onset of cognitive symptoms. Synaptic degeneration is positively related to the severity of dementia (DeKosky & Scheff, 1990; Jacobsen et al., 2006; Reddy et al., 2005; Scheff et al., 2006; Terry et al., 1991). Several synaptic proteins have been reported to be decreased in brain specimens from AD patients (Bereczki et al., 2016, 2018; Goetzl et al., 2016; Qin et al., 2004; Reddy et al., 2005). However, the molecular mechanisms underlying synaptic dysfunction in AD remain elusive.

Synapsin Ⅰ is a neuron‐specific phosphoprotein localized to the cytoplasmic surface of synaptic vesicles (SVs). It acts as the key regulator of SV dynamics in presynaptic terminals. Under resting conditions, synapsin Ⅰ clusters SVs in the reserve pool by interacting with phospholipids and the F‐actin cytoskeleton. Upon stimulation, synapsin Ⅰ is phosphorylated at Ser9 and dissociates from SVs. After the stimulus, synapsin Ⅰ is dephosphorylated and reclusters SVs in the bouton (Cesca et al., 2010). Thus, the normal function of synapsin Ⅰ is required for the recycling of SVs in the presynaptic terminal. In AD patients, the expression of synapsin Ⅰ is decreased in the brain, which is accompanied by synaptic dysfunction (Qin et al., 2004), indicating that the dysfunction of synapsin Ⅰ may contribute to synaptic dysfunction in AD.

Mammalian asparagine endopeptidase (AEP) is a lysosomal cysteine protease that cleaves protein substrates on the C‐terminal side of asparagine residues (Chen et al., 1998; Chen et al., 1998). AEP is activated by sequential removal of C‐ and N‐terminal propeptides under acidic conditions (Li et al., 2003). Recently, we reported that AEP is activated in the brain in an age‐dependent manner and cleaves amyloid precursor protein (APP) and tau, promoting the deposition of amyloid‐β (Aβ) and tau during the onset of AD (Zhang et al., 2014, 2015). In this report, we show that AEP cleaves synapsin Ⅰ at N82 both *in vitro* and *in vivo*, generating the synapsin Ⅰ (83–705) fragment (designated C83 fragment). This fragment induces synaptic dysfunction both *in vitro* and *in vivo*. Overexpression of AEP in the hippocampus of wild‐type mice results in the production of synapsin Ⅰ C83 and synaptic dysfunction. Furthermore, the expression of the synapsin Ⅰ C83 fragment in tau P301S transgenic mice or wild‐type mice promotes synaptic dysfunction and cognitive impairment. Hence, our results indicate that AEP‐mediated fragmentation of synapsin Ⅰ contributes to synaptic dysfunction in AD.

## 2 RESULTS

### 2.1 Synapsin Ⅰ is a substrate of AEP

**FIGURE 1:**
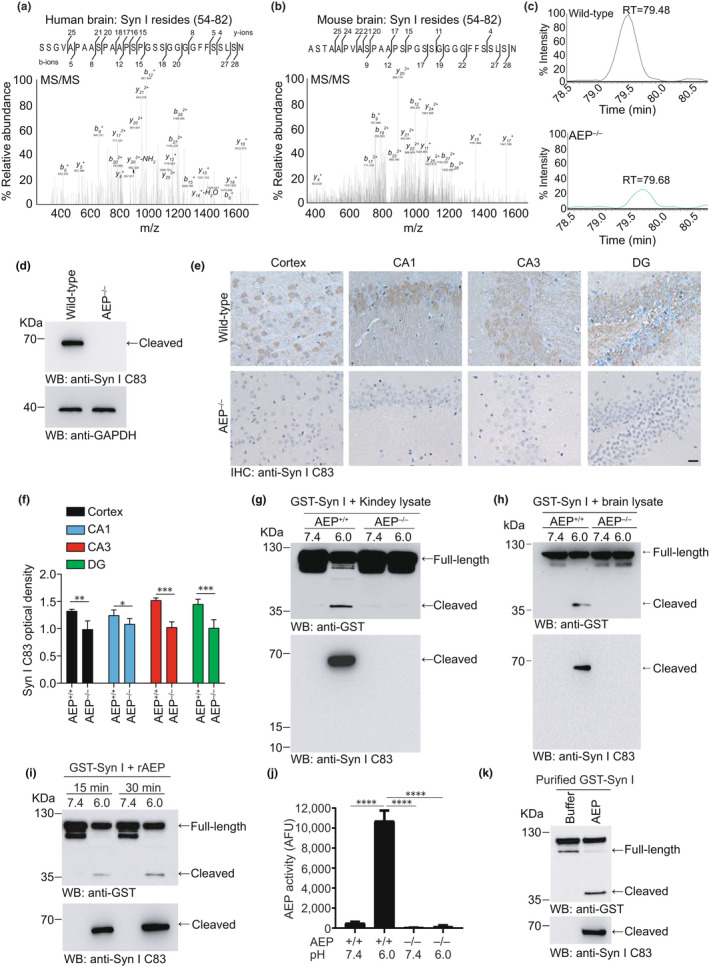
AEP cleaves synapsin Ⅰ *in vitro*. (a) MS/MS spectrum showing the presence of synapsin Ⅰ (54–82) peptide in brain samples from subjects with AD. (b) MS/MS spectrum showing the presence of synapsin Ⅰ (54–82) peptide in brain samples from wild‐type mice. (c) Representative extracted ion chromatograms for synapsin Ⅰ (54–82) peptide from wild‐type and AEP^–/–^ mouse brain samples. (d) Western blot detection of synapsin Ⅰ C83 fragment in the wild‐type and AEP^–/–^ mouse brain lysates. (e, f) Immunostaining of synapsin Ⅰ C83 fragments in brain slices of wild‐type and AEP^–/–^ mice (e), and Syn I C83 optical density (f). DG: dentate gyrus. Scale bar, 20 μm. (g) Synapsin Ⅰ cleavage assay. GST‐synapsin Ⅰ was incubated with kidney lysates from wild‐type and AEP^–/–^ mice at pH 7.4 or pH 6.0 at 37°C for 30 min, respectively. Western blot shows that synapsin Ⅰ was cleaved at pH 6.0 by wild‐type kidney lysates when AEP was activated (j) (*n* = 3). (h) Synapsin Ⅰ cleavage assay. GST‐synapsin Ⅰ was incubated with brain lysates from wild‐type and AEP^–/–^ mice at pH 7.4 or pH 6.0 at 37°C for 30 min, respectively. Western blot shows that synapsin Ⅰ was cleaved at pH 6.0 by wild‐type brain lysates. (i) Western blot showing the time‐dependent cleavage of synapsin Ⅰ by purified activated recombinant AEP (rAEP). (k) Western blot showing the cleavage of purified GST‐synapsin Ⅰ by AEP. Syn I, synapsin I. Data are mean ± SEM; ^*^ *p* < 0.005, ^**^ *p* < 0.001, ^***^ *p* < 0.001, ^****^ *p* < 0.0001

To investigate the molecular mechanisms of synaptic dysfunction, we analyzed brain tissues from AD patients by mass spectrometry. Interestingly, a fragment ending at N82 of synapsin Ⅰ was found in the AD brain (Figure 1a). Since AEP is the only protease that specifically cleaves on the C‐terminal side of asparagine residues, we speculate that this fragment is generated by AEP‐mediated cleavage. To verify that synapsin Ⅰ is cleaved by AEP, we performed mass spectrometry with brain tissues from wild‐type and AEP knockout (AEP^–/–^) mice. The same fragment of synapsin Ⅰ was identified in brain tissue lysates from wild‐type mice (Figure 1b). Comparative label‐free proteomic analysis of wild‐type mice and AEP^–/–^ mice revealed that the signal of this fragment was much lower in AEP^–/–^ mouse brain extracts (Figure 1c). These results indicate that AEP may be responsible for the fragmentation of synapsin Ⅰ in the brain. To further investigate the presence of the synapsin Ⅰ C83 fragment in the brain, we generated an antibody that specifically recognizes the AEP‐generated C83 fragment of synapsin Ⅰ (anti‐synapsin Ⅰ C83 antibody) but not full‐length synapsin Ⅰ. Using this antibody, we detected the presence of the synapsin Ⅰ C83 fragment in wild‐type mice but not in AEP^–/–^ mice by Western blotting and immunohistochemistry (Figure 1d–f), supporting the specificity of this antibody for the AEP‐generated synapsin Ⅰ fragment.

To confirm the cleavage of synapsin Ⅰ by AEP, we performed an *in vitro* cleavage assay by incubating GST‐synapsin Ⅰ with kidney lysates prepared from wild‐type and AEP^–/–^ mice at pH 7.4 or 6.0. AEP was activated at pH 6.0 and generated an N‐terminal synapsin Ⅰ fragment that was detected with the anti‐GST antibody and a C‐terminal fragment that was detected with the anti‐synapsin Ⅰ C83 antibody (Figure 1g). The enzymatic activity of AEP in kidney lysates was confirmed by an enzyme activity assay (Figure 1j). GST‐synapsin Ⅰ can also be cleaved by brain lysate from wild‐type mice, but not that from AEP^–/–^ mice at pH 6.0 (Figure 1h). To further explore the cleavage of synapsin Ⅰ by AEP, we incubated the active AEP enzyme with GST‐synapsin Ⅰ for 15 min and 30 min, respectively. Western blot analysis showed that synapsin Ⅰ was fragmented in the presence of AEP in a time‐dependent manner (Figure 1i). To exclude the potential effect of other cellular components in HEK293 cell lysates, we incubated purified GST‐synapsin Ⅰ with active recombinant AEP. As expected, purified GST‐synapsin Ⅰ was also potently cleaved by AEP (Figure 1k). Overall, these results indicate that synapsin Ⅰ is a substrate of AEP.

### 2.2 AEP cleaves synapsin Ⅰ at N82

**FIGURE 2:**
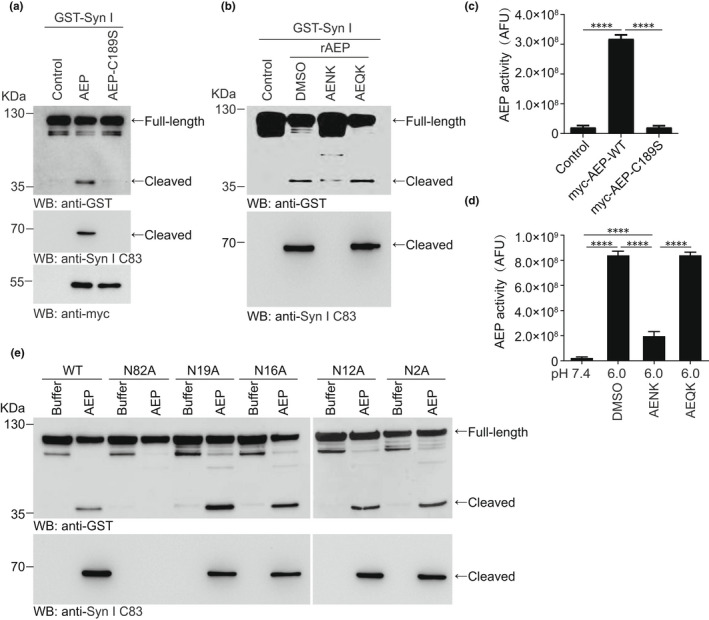
AEP cleaves synapsin Ⅰ at N82. (a) Synapsin Ⅰ cleavage by wild‐type and mutant AEP. Wild‐type AEP but not loss‐of‐function mutant AEP cleaved GST‐synapsin Ⅰ. (b) The cleavage of synapsin Ⅰ was inhibited by AEP inhibitor AENK but not by AEQK. (c, d) AEP activity assay showing that the C189S mutant of AEP is not active (c), and AENK inhibits AEP activity (d). (e) The cleavage of mutant synapsin Ⅰ by AEP. Fragmentation of synapsin Ⅰ was analyzed by Western blot after GST‐synapsin Ⅰ wild‐type, N82A, N19A, N16A, N12A, and N2A mutants were incubated with recombinant AEP. ^****^ *p* < 0.0001

To confirm that AEP specifically cleaves synapsin Ⅰ, we co‐transfected GST‐synapsin Ⅰ and myc‐AEP or myc‐AEP C189S mutant into HEK293 cells. Wild‐type AEP strongly induced synapsin Ⅰ fragmentation, while the AEP C189S mutant, whose protease activity is abolished (Li et al., 2003), could not trigger synapsin Ⅰ cleavage (Figure 2a). Moreover, this cleavage process was suppressed by the AEP inhibitor AENK, whereas the inactive control AEQK had no effect (Figure 2b). The enzymatic activity of AEP was validated by a fluorescent substrate cleavage assay (Figure 2c,d). These findings indicate that the synapsin Ⅰ C83 fragment is specifically generated by AEP. The results of mass spectrometric analysis of brain tissues from AD patients and mice suggest that AEP cleaves synapsin Ⅰ at N82. AEP selectively cleaves its substrates on the C‐terminal side of asparagine residues. The N‐terminus of synapsin Ⅰ contains 5 asparagine residues (N2, N12, N16, N19, and N82). We generated synapsin Ⅰ point mutants with these asparagines substituted with alanines. Generation of the synapsin Ⅰ fragment was completely abolished in the N82A mutant, but not in the N2A, N12A, N16A, or N19A mutant (Figure 2e). These results indicate that AEP specifically cleaves synapsin Ⅰ at N82, generating the C83 fragment.

### 2.3 AEP is upregulated during aging and generates the synapsin Ⅰ C83 fragment in AD brain

**FIGURE 3:**
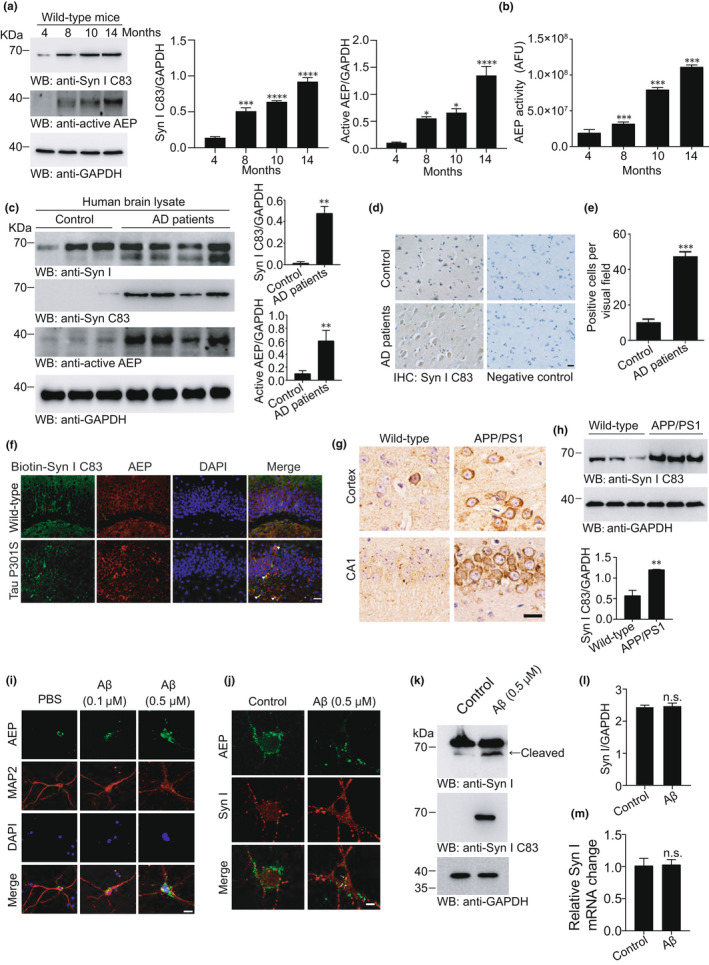
AEP is activated and cleaves synapsin Ⅰ in the AD brain. (a) Western blot analysis of synapsin Ⅰ and active AEP in 4‐, 8‐, 10‐, and 14‐month‐old wild‐type mice brain, bar graphs are quantitative analysis of Syn I C83 and AEP. (b) AEP activity assay showing the age‐dependent activation of AEP in mouse brain. (c) Western blot detection of synapsin Ⅰ C83 fragment in human brain samples from subjects with AD and age‐matched controls, bar graphs are quantitative analysis of Syn I C83 and AEP. (d, e) Immunostaining of synapsin Ⅰ C83 fragments in brain slices of AD patients and age‐matched control. Scale bar, 20 μm. (f) Immunostaining showing the colocalization of synapsin Ⅰ C83 fragment with AEP in tau P301S mouse brain slices. Scale bar, 20 μm. (g) Immunostaining of synapsin Ⅰ C83 fragments in slices of wild‐type and APP/PS1 mice. Scale bar, 20 μm. (h) Western blot detection of synapsin Ⅰ C83 fragment in hippocampus brain lysates of wild‐type and APP/PS1 mice. bar graphs are quantitative analysis of Syn I C83. (i) Immunostaining shows that Aβ induces the transport of AEP from soma to neurite in the primary neuron. Scale bar, 20 μm. (j) AEP colocalizes with synapsin Ⅰ after the neurons were challenged with Aβ oligomers. Scale bar, 10 μm. (k, l) Synapsin Ⅰ C83 fragment was detected by Western blot after the neurons were treated with Aβ oligomers for 24 h. Bar graphs are quantification of full‐length Synapsin I (l). (m) The mRNA levels of synapsin Ⅰ detected by RT‐PCR in the presence or absence of Aβ oligomers. ns, no significance. Data are mean ± SEM; ^**^ *p* < 0.001, ^***^ *p* < 0.001, ^****^ *p* < 0.0001

Aging is the major risk factor for AD (Guerreiro & Bras, 2015). AEP is activated during aging as the internal milieu of the brain gradually acidifies (Eugenin et al., 2016; Pirchl et al., 2006; Yates et al., 1990). We investigated the appearance of the AEP‐generated synapsin Ⅰ C83 fragment in the mouse brain at different ages. The anti‐synapsin Ⅰ C83 antibody detected the C83 fragment in brain tissue lysates from 4‐, 8‐, 10‐, and 14‐month‐old mice, and the amount of the C83 fragment increased with age (Figure 3a). The AEP activity assay showed that AEP was activated in an age‐dependent manner in the mouse brain (Figure 3b). We further verified the presence of the synapsin Ⅰ C83 fragment in the human AD brain using Western blotting and immunohistochemistry. Synapsin Ⅰ C83 was abundant in human AD brains but was present at much lower levels in the brains of age‐matched controls (Figure 3c–e). We also detected the presence of the synapsin Ⅰ C83 fragment in the hippocampal CA1 region in the tau P301S transgenic mice, but little was detected in brain sections from age‐matched nontransgenic control mice. Furthermore, immunofluorescence staining showed that AEP colocalized with synapsin Ⅰ C83 (Figure 3f). CCAAT/enhancer‐binding protein β (C/EBPβ) is the transcriptional factor that promotes the expression of AEP in the brain of AD patients and AD mouse models (Wang et al., 2018, 2019; Wang et al., 2018). We found that the levels of C/EBPβ, phosphorylated C/EBPβ, active AEP, and synapsin Ⅰ C83 were much higher in 5‐month‐old tau P301S mice when compared with those in age‐matched wild‐type mice (Figure S1a,b). Western blot and immunohistochemistry confirmed that synapsin Ⅰ C83 fragment was also abundant in brain lysates from APP/PS1 transgenic mice, a well‐established animal model of AD (Figure 3g,h). Therefore, AEP is activated during aging and cleaves synapsin Ⅰ in the brain of tau P301S mice and APP/PS1 mice.

Asparagine endopeptidase is a lysosomal protease that leaks into the cytoplasm in neurodegenerative diseases (Basurto‐Islas et al., 2013; Zhang et al., 2017). To explore the interaction between AEP and synapsin Ⅰ under pathological conditions, we detected the distribution of AEP in neurons treated with Aβ oligomers. AEP was localized mainly in the neuronal soma, but after neurons were challenged with Aβ, it translocated from the neuronal soma to the neurite (Figure 3i), where it colocalized with synapsin Ⅰ (Figure 3j). Aβ also induced the generation of the synapsin Ⅰ C83 fragment in primary neurons (Figure 3k) but did not change the levels of total Synapsin Ⅰ protein and mRNA (Figure 3l,m). These results indicate that AEP translocates to neurites under pathological conditions and cleaves synapsin I.

### 2.4 Synapsin Ⅰ C83 fragment induces synaptic dysfunction *in vitro*

**FIGURE 4:**
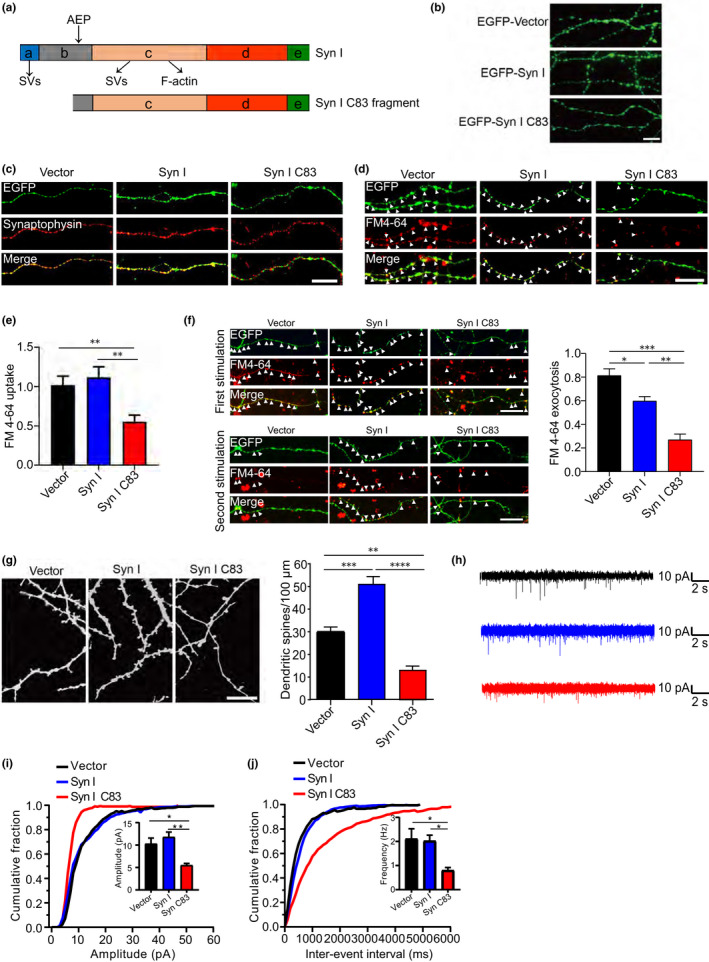
Overexpression of the synapsin I fragmentation induces synaptic dysfunction. (a) Schematic diagram of synapsin I domain and its cleavage by AEP. The binding sites of synapsin Ⅰ to SVs and F‐actin are shown. (b) Fluorescent micrographs showing the distribution of full‐length synapsin Ⅰ and synapsin Ⅰ C83 fragment. Scale bar, 20 μm. (c) Immunostaining showing the colocalization of full‐length and synapsin Ⅰ C83 fragment with synaptophysin. Scale bar, 20 μm. (d, e) FM4‐64 uptake assay. The uptake of FM4‐64 is decreased in neurons expressing synapsin Ⅰ C83 fragment when compared with neurons expressing full‐length synapsin Ⅰ. Scale bar, 20 μm. (f) FM4‐64 releasing assay. The release of FM4‐64 was decreased in neurons expressing synapsin Ⅰ C83 fragment when compared with neurons expressing full‐length synapsin Ⅰ. Scale bar, 20 μm. (g) Dil staining of dendritic spines. The density of dendritic spines in neurons expressing synapsin Ⅰ C83 is lower than that in neurons expressing full‐length synapsin Ⅰ. Scale bar, 10 μm. Bar graphs are the quantification of the dendritic densities (*n* = 10). (h) Representative traces of mEACs recorded from isolated hippocampal neurons expression EGFP‐vector, EGFP‐synapsin I, and EGFP‐synapsin Ⅰ C83. (i) Cumulative plots and mean values (inset) of mEACs amplitude in isolated hippocampal neurons expression vector, synapsin Ⅰ, and synapsin Ⅰ C83. (j) Cumulative plots of mEACs inter‐event interval and mean values (inset) of mEACs frequency in isolated hippocampal neurons in various conditions. Kolmogorov‐Smirnov test in cumulative plots (*n* = 4–6 per group). Data are mean ± SEM; one‐way ANOVA; ^*^ *p* < 0.005, ^**^ *p* < 0.001, ^***^ *p* < 0.001, ^****^ *p* < 0.0001

Synapsin Ⅰ regulates neurotransmitter release by interacting with lipids and the actin cytoskeleton to cluster the SVs in the reserve pool (Cesca et al., 2010). A schematic diagram of synapsin Ⅰ and its cleavage by AEP is shown in (Figure 4a). To assess whether AEP‐mediated cleavage of synapsin Ⅰ affects the normal function of synapsin Ⅰ in neurons, we infected primary neurons with adeno‐associated viruses (AAVs) encoding EGFP, EGFP‐synapsin Ⅰ, and EGFP‐synapsin Ⅰ C83, respectively. Synapsin Ⅰ was distributed in a punctate pattern, whereas synapsin Ⅰ C83 was more diffusely distributed (Figure 4b). Immunofluorescence staining showed that full‐length synapsin Ⅰ colocalized with synaptophysin, a membrane protein localized to SVs, whereas synapsin Ⅰ C83 did not (Figure 4c). These results imply that the C83 fragment does not bind to SVs as does the full‐length protein.

Next, we investigated whether the synapsin Ⅰ C83 fragment impairs the recycling of SVs in neurons through an FM4‐64 labeling assay. FM4‐64 labeling in boutons expressing synapsin Ⅰ C83 was reduced by 45% compared with that in boutons overexpressing full‐length synapsin Ⅰ (Figure 4d,e). Furthermore, the release of FM4‐64 was also impaired in the presence of synapsin Ⅰ C83 (Figure 4f). In addition, DiI staining showed that the density of dendritic spines in neurons expressing synapsin Ⅰ C83 was decreased compared with that in neurons expressing full‐length synapsin Ⅰ (Figure 4g), indicating that the synapsin Ⅰ C83 fragment induces spine degeneration. These results indicate that the synapsin Ⅰ C83 fragment is abnormally distributed, impairs SV recycling, and induces dendritic spine degeneration. To investigate whether the synapsin Ⅰ C83 fragment causes presynaptic defects, we recorded miniature excitatory autaptic currents (mEACs) from individually inhabited neurons grown on collagen/poly‐D‐lysine (PDL) islands (Figure 4h). The frequency and amplitude of mEACs were significantly lower in neurons expressing synapsin Ⅰ C83 than in neurons expressing vector and full‐length synapsin Ⅰ (Figure 4i,j). Collectively, the above results suggest that the synapsin Ⅰ C83 fragment interferes with synaptic transmission.

### 2.5 Overexpression of AEP induces synaptic dysfunction and cognitive impairment *in vivo*

**FIGURE 5:**
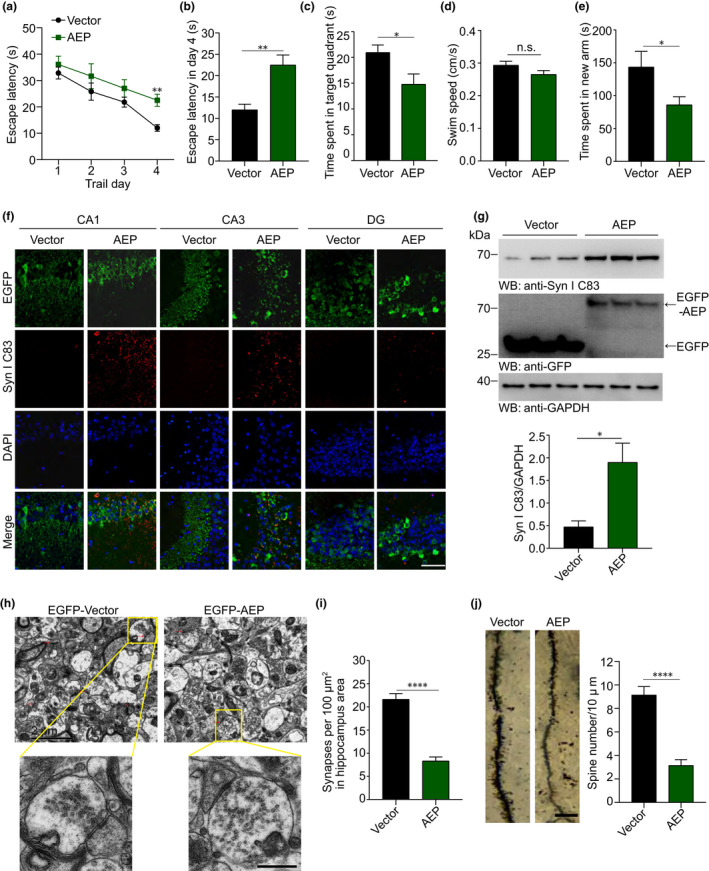
Overexpression of AEP induces synapsin Ⅰ fragmentation and synaptic dysfunction in wild‐type mice. (a, b) Morris water maze test as escape latency (s) and escapes latency in day 4 (s) (*n* = 8). (c) Probe trial of Morris water maze test (*n* = 8). (d) Swim speed of mice injected with AAVs encoding EGFP and EGFP‐AEP (*n* = 8). (e) Y‐maze test as time spent in new arms (*n* = 8). (f) Immunofluorescent staining of synapsin Ⅰ C83 fragment in the hippocampus from mice injected with AAVs encoding EGFP and EGFP‐AEP. Scale bar, 50 μm. (g) Western blots showing the expression of synapsin Ⅰ C83 fragment in the hippocampus of mice injected with AAVs encoding EGFP and EGFP‐AEP (*n* = 3). (h, i) Electron microscopy of the synapses. Red arrows indicate synapses. Scale bar, 2 μm (upper panel), 500 nm (lower panel) (*n* = 8). (j) Golgi staining of the spines from the hippocampal area. Scale bar, 20 μm (*n* = 8). Data are mean ± SEM; *t*‐test; ^*^ *p* < 0.05, ^**^ *p* < 0.01, ^****^ *p* < 0.0001

We next assessed the impacts of AEP on synaptic and cognitive function *in vivo*. AAVs encoding EGFP and EGFP‐AEP were injected separately into the hippocampal area of 2‐month‐old wild‐type mice. Three months later, we assessed the effect of AEP overexpression on spatial learning and memory via the Morris water maze test. During the training phase, the latency to find the platform was gradually decreased in all mice, indicating a learning effect. However, the learning ability of mice overexpressing AEP was substantially impaired (Figure 5a,b). In the probe trial, mice expressing AEP showed a decreased time in the target quadrant, suggesting impaired memory function (Figure 5c). The swim speeds were comparable among the mice (Figure 5d), suggesting that overexpression of AEP does not affect motor function. Similarly, in the Y‐maze test, mice expressing AEP spent less time in the new arm (Figure 5e). These results indicate that overexpression of AEP in the mouse brain induces learning and memory impairments.

Immunofluorescence staining showed stronger synapsin Ⅰ C83 signals in the CA1 and CA3 regions of the hippocampus in mice overexpressing AEP than in control mice. Moreover, EGFP‐AEP colocalized with synapsin C83 fragments (Figure 5f). Western blot analysis showed that the abundance of the synapsin Ⅰ C83 fragment was increased in hippocampal lysates from mice expressing AEP compared with control mice (Figure 5g). These results suggest that overexpression of AEP induces the production of the synapsin Ⅰ C83 fragment *in vivo*. Electron microscopy analysis of the hippocampal area showed that the density of synapses in the hippocampus of mice injected with AAV‐EGFP‐AEP was reduced compared with that in the control group. Furthermore, the distribution of SVs was more diffuse compared with the control group (Figure 5h,i), indicating that the overexpression of AEP results in a reduced ability for SV clustering in the reserve pool. In addition, Golgi staining revealed that AEP induced the loss of dendritic spines in wild‐type mice (Figure 5j). Hence, overexpression of AEP induces synaptic dysfunction and cognitive impairment *in vivo*.

### 2.6 Synapsin Ⅰ C83 fragment induces synaptic dysfunction and cognitive impairment in tau P301S transgenic mice

**FIGURE 6:**
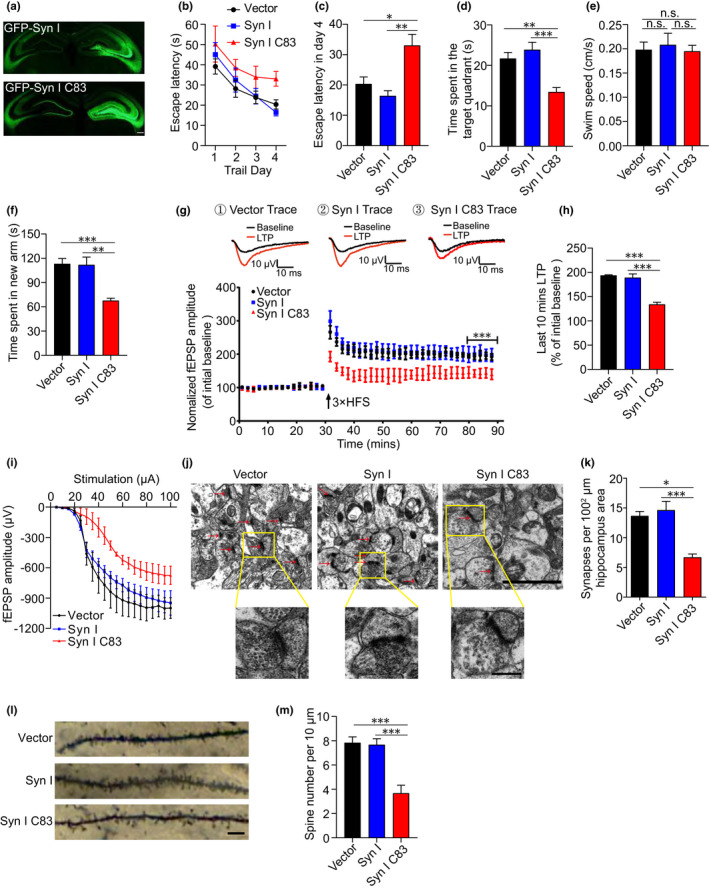
Overexpression of synapsin Ⅰ C83 fragment induces synaptic dysfunction and cognitive impairment in tau P301S mice. (a) The expression of synapsin Ⅰ and synapsin Ⅰ C83 in the hippocampal area of tau P301S mice. Scale bar, 100 μm. (b, c) Morris water maze test as escape latency (s) and escapes latency in day 4 (s) (*n* = 8). (d) Probe trial of Morris water maze test (*n* = 8). (e) Swim speed of mice injected with AAVs encoding EGFP‐vector, EGFP‐synapsin Ⅰ FL, and EGFP‐synapsin Ⅰ C83 (*n* = 8). (f) Y‐maze test as time spent in new arms (*n* = 8). (g) The amplitude of fEPSP after HFS recorded on hippocampal slices. Arrow indicates HFS onset. Shown traces are representative fEPSPs of 3 samples recorded before and after LTP induction. (h) Quantitative analysis for normalized fEPSPs 50–60 min after HFS (*n* = 3 mice per group). (i) The input/output (I/O) curves of fEPSP. (j, k) Electron microscopy of the synapses. Red arrows indicate synapses (*n* = 8). Scale bar, 2 μm (upper panel), 500 nm (lower panel). (l, m) Golgi staining showing the spines from the hippocampal area (*n* = 8). Scale bar, 20 μm. Data are mean ± SEM; one‐way ANOVA; ^*^ *p* < 0.005, ^**^ *p* < 0.001, ^***^ *p* < 0.001

We further investigated the impact of the AEP‐generated synapsin Ⅰ C83 fragment on synaptic dysfunction and cognitive impairment in tau P301S transgenic mice, a mouse model of tauopathy, which is related to AD and related disorders. The mice express human mutant tau P301S and develop widespread neurofibrillary tangle‐like inclusions in the brain, accompanied by synaptic dysfunction and behavioral impairment (Yoshiyama et al., 2007). We found that the 5‐month‐old tau P301S mice showed cognitive impairment in the Morris water maze test and Y‐maze test (Figure S2a–e). AAVs encoding EGFP, EGFP‐synapsin Ⅰ, and EGFP‐synapsin Ⅰ C83 were injected separately into the hippocampal region of 2‐month‐old tau P301S mice. Three months later, we observed strong green fluorescence signals in the hippocampal area in all mice (Figure 6a). The effect of the overexpression of the synapsin Ⅰ C83 fragment on the spatial memory of tau P301S transgenic mice was assessed via the Morris water maze test. During the training phase, the latency to find the platform was gradually decreased in tau P301S transgenic mice expressing EGFP. However, the learning ability of mice expressing synapsin Ⅰ C83 was substantially impaired (Figure 6b,c). In the probe trial, mice expressing synapsin Ⅰ C83 showed a decreased time in the target quadrant, indicating that their learning and memory abilities were impaired (Figure 6d). The swim speed was comparable among the mice (Figure 6e), indicating that overexpression of full‐length synapsin Ⅰ or its fragment does not affect motor function. Consistent with the water maze test results, in the Y‐maze test, mice expressing synapsin Ⅰ C83 spent less time in the new arm (Figure 6f). The long‐term potentiation (LTP) of field excitatory postsynaptic potentials (fEPSPs) in the hippocampus is believed to be the basis of learning and memory (Fedulov et al., 2007; Nicoll, 2017). We observed that LTP was diminished in mice expressing synapsin Ⅰ C83 compared with mice expressing GFP or full‐length synapsin Ⅰ (Figure 6g). The average amplitude of fEPSPs was lower in mice expressing synapsin Ⅰ C83 than in those expressing EGFP or full‐length synapsin Ⅰ (Figure 6h). The I/O curve showed that the fEPSP response of Syn I C83 mice was weaker than that of the other groups (Figure 6i). These results indicate that overexpression of the synapsin Ⅰ C83 fragment induces synaptic dysfunction and cognitive impairment in tau P301S transgenic mice. Electron microscopy analysis of mouse hippocampal slices showed that the synaptic density was lower in mice expressing synapsin Ⅰ C83 than in mice expressing GFP or full‐length synapsin Ⅰ (Figure 6j,k). In addition, Golgi staining showed that the synapsin Ⅰ C83 fragment induced loss of dendritic spines in AD model mice (Figure 6l,m). In summary, the AEP‐generated synapsin Ⅰ C83 fragment induces synaptic dysfunction and cognitive impairment in tau P301S mice.

### 2.7 Synapsin Ⅰ C83 fragment induces synaptic dysfunction and cognitive impairment in wild‐type mice

To investigate the direct impact of synapsin Ⅰ C83 fragment on the synaptic dysfunction and cognitive impairment, AAVs encoding EGFP, EGFP‐synapsin Ⅰ, and EGFP‐synapsin Ⅰ C83 were injected into the bilateral CA1 hippocampal region of 2‐month‐old wild‐type mice. 2.5 months later, the spatial memory was assessed via the Morris water maze test. During the training phase, the latency to find the platform was gradually decreased in wild‐type mice expressing EGFP. However, the learning ability of mice expressing synapsin Ⅰ C83 was substantially impaired (Figure S3a,b). In the probe trial, mice expressing synapsin Ⅰ C83 showed a decreased time in the target quadrant, indicating that their learning and memory abilities were impaired (Figure S3c). The swim speed was comparable among the mice (Figure S3d). In the Y‐maze test, mice expressing synapsin Ⅰ C83 spent less time in the new arm (Figure S3e). Furthermore, the LTP was diminished in mice expressing synapsin Ⅰ C83 compared with mice expressing GFP or full‐length synapsin Ⅰ (Figure S3f). The average amplitude of fEPSPs was lower in mice expressing synapsin Ⅰ C83 than in those expressing EGFP or full‐length synapsin Ⅰ (Figure S3g). Hence, the synapsin Ⅰ C83 fragment induces synaptic dysfunction and cognitive impairment in wild‐type mice.

## 3 DISCUSSION

In the present study, we identified that the synaptic protein synapsin Ⅰ is a substrate of the cysteine protease AEP. AEP is activated and cleaves synapsin Ⅰ in an age‐dependent manner in the mouse brain and human AD brain. The AEP‐generated synapsin Ⅰ C83 fragment exhibits a reduced ability to localize at SVs, disrupts vesicle recycling, and causes presynaptic defects. Overexpression of AEP or the AEP‐generated synapsin Ⅰ C83 fragment in mice induces synaptic dysfunction and cognitive impairment. Therefore, the synapsin Ⅰ C83 fragment mediates synaptic dysfunction and cognitive impairment.

Neurotransmitters are stored in SVs in the axon terminal. In response to an action potential, SVs fuse with the presynaptic membrane, thus releasing neurotransmitters into the synaptic cleft. Synapsin Ⅰ is an SV‐associated protein that acts as a link between extracellular stimuli and intracellular signaling events. Under resting conditions, synapsin Ⅰ clusters SVs in the reserve pool by interacting with phospholipids and F‐actin. Upon stimulation, synapsin Ⅰ is phosphorylated and dissociates from the reserve pool, and SVs move close to the readily releasable pool. Deletion of synapsin Ⅰ induces synaptic dysfunction and cognitive impairment in mice (Corradi et al., 2008; Ryan et al., 1996). We found that overexpression of full‐length synapsin I in primary neurons increased the density of dendritic spines. This is consistent with the previous reports that synapsin I promotes the outgrowth of neurites and maintains the physiological function of neurons (Cesca et al., 2010; Fornasiero et al., 2010). However, AEP cleaves synapsin Ⅰ and generates the synapsin Ⅰ C83 fragment, which induces spine degeneration and impairs SV recycling and synaptic transmission. We found that overexpression of full‐length synapsin I led to synaptic dysfunction and cognitive impairment in wild‐type mice (Figure S2), indicating that massive overexpression of synapsin I exerts detrimental effects on normal synapses *in vivo*. Synaptic impairments precede the formation of tangles in tau P301S mice (Yoshiyama et al., 2007). We found that overexpression of full‐length synapsin I did not exacerbate synaptic dysfunction in tau P301S mice, even though the full‐length synapsin I could be cleaved to the synapsin I C83 fragment by endogenous AEP. These results indicate that the protective effect of full‐length synapsin I counteracted the detrimental effect of the synapsin I C83 fragment in tau P301S mice.

Synapsin Ⅰ consists of five domains named A‐E. Domain A is a short N‐terminal domain that contains the major phosphorylation site at Ser9, which can be phosphorylated by protein kinase A (PKA) and dephosphorylated by protein phosphatase 2A (PP2A). The phosphorylation status of Ser9 governs its reversible association with SVs. The basal level of Ser9 phosphorylation is low and allows clustering of SVs in the reserve pool. Upon stimulation, Ser9 is phosphorylated by PKA, which dissociates synapsin Ⅰ from SVs, triggering the release of neurotransmitters. After the stimulus, synapsin Ⅰ is dephosphorylated and reclusters on SVs (Cesca et al., 2010; Song & Augustine, 2015). Thus, the phosphorylation‐dephosphorylation cycle of synapsin Ⅰ regulates synaptic function. AEP‐mediated cleavage of synapsin Ⅰ generates the C83 fragment, which does not contain the A domain and part of the B domain. Conceivably, the C83 fragment in the AD brain cannot undergo the normal phosphorylation‐dephosphorylation cycle, inducing synaptic dysfunction. This hypothesis is supported by the finding that overexpression of the C83 fragment induces synaptic dysfunction both *in vitro* and *in vivo*.

Here we show that AEP‐mediated cleavage of synapsin Ⅰ induces synaptic dysfunction and accelerates the pathological process of AD in mice. Overexpression of AEP in the hippocampus of wild‐type mice induces synaptic dysfunction and cognitive impairment. Furthermore, the expression of synapsin Ⅰ C83 in the tau P301S transgenic mouse model of tauopathy and wild‐type mice shows similar pathological effects. Taken together, our results indicate that AEP cleaves synapsin Ⅰ and contributes to synaptic dysfunction in AD. Considering the pivotal role of AEP in synaptic dysfunction, inhibition of this protease may be a novel and promising therapeutic intervention for AD.

## 4 MATERIALS AND METHODS

### 4.1 Mice

Tau P301S mice on a C57BL/6J background (line PS19) and wild‐type C57BL/6J mice were from The Jackson Laboratory (stock number: 008169 and 000664, respectively). The AEP knockout mice on a mixed 129/Ola and C57BL/6 background were generated as reported (Shirahama‐Noda et al., 2003). Animal care and handling were performed according to the Declaration of Helsinki and guidelines of Renmin Hospital, Wuhan University. The sample size was determined by Power and Precision (Biostat). Male and female animals were evenly assigned to each group. The investigators were blinded to the group allocation during the animal experiments. The protocol was reviewed and approved by the Animal Care and Use Committee of Renmin Hospital of Wuhan University. The IACUC approval number is 20180101.

### 4.2 Antibodies and reagents

Antibodies to the following targets were used: Synapsin Ⅰ (Cell Signaling Technology, 5297), synaptophysin (Proteintech, 17785–1‐AP), AEP (Cell Signaling Technology, 93627), GFP (Proteintech, 66002–1‐Ig), GST (Proteintech, 66001–2‐Ig), myc (Proteintech, 16286–1‐AP), GAPDH (Proteintech, 60004–1‐Ig), HRP‐conjugated anti‐mouse IgG (BIO‐RAD, 170–6516), HRP‐conjugated anti‐rabbit IgG (BIO‐RAD, 170–6515), Alexa Fluor 594‐conjugated goat anti‐rabbit IgG (Invitrogen, A‐11012), Alexa Fluor 594‐conjugated goat anti‐mouse IgG (Invitrogen, A‐11005), Alexa Fluor 488‐conjugated goat anti‐rabbit IgG (Invitrogen, A11034). Dil (Thermo Fisher Scientific, 1975524), recombinant AEP (Sino Biological, Beijing, China), FD Rapid Golgi Stain Kit (FD Neuro Technologies, Inc, PK401), AEP substrate Z‐Ala‐Ala‐Asn‐AMC (Bachem), ProLong^TM^ gold antifade mountant with DAPI (Invitrogen, P36941).

### 4.3 Human tissue samples

Postmortem brain samples of AD cases and age‐matched control cases were from the Emory Alzheimer's Disease Research Center. The average ages of the AD patients and control subjects were 65.4 and 63.2, respectively. Hippocampal brain samples were used in this study. The stages of the samples were Braak stage 4–6. All the AD cases were confirmed by pathological diagnosis. The staging of AD pathology was determined using the method described previously (Braak et al., 2006). The study was approved by the Biospecimen Committee.

### 4.4 Synapsin Ⅰ cleavage assay and AEP activity assay

Synapsin Ⅰ cleavage assay and AEP activity assay were conducted as described previously (Zhang et al., 2014). Briefly, HEK293 cells were transfected with GST‐synapsin Ⅰ plasmids. 48 h later, cells were lysed in AEP buffer (50‐mM sodium citrate, 5‐mM DTT, 0.1% CHAPS and pH 5.5, 0.5% Triton X‐100), centrifuged for 15 min at 12,000 rpm at 4°C. Supernatants were incubated with AEP (5 μg/ml) at pH 6.0 or 7.4 at 37°C for 15 min and 30 min, respectively. All samples were analyzed by Western blot. For the AEP activity assay, cell lysates or tissue homogenates were incubated in a 200‐μl assay buffer (20‐mM citric acid, 60‐mM Na_2_HPO_4_, 1‐mM EDTA, 0.1% CHAPS, and 1‐mM DTT, pH 6.0) containing 20‐μM AEP substrate Z‐Ala‐Ala‐Asn‐AMC. AMC released by enzymatic cleavage was quantified by measuring at 460 nm in a fluorescence plate reader at 37°C for 1 h in kinetic mode for 5 min.

### 4.5 Mass spectrometry analysis

AEP^–/–^ mice and age‐matched wild‐type mice brain lysate samples were in‐gel digested with trypsin. The samples were resuspended in loading buffer (0.1% formic acid, 0.03% trifluoroacetic acid, 1% acetonitrile) and loaded onto a 20 cm nano‐high performance liquid chromatography column (internal diameter 100 µm) packed with Reprosil‐Pur 120 C18‐AQ 1.9 µm beads (Dr. Maisch) and eluted over a 2 h 4–80% buffer B reverse phase‐gradient (buffer A: 0.1% formic acid and 1% acetonitrile in water; buffer B: 0.1% formic acid in acetonitrile) generated by a NanoAcquity UPLC system (Waters Corporation). Samples were ionized on a hybrid LTQ XL Orbitrap mass spectrometer (Thermo) using a 2.0 kV electrospray ionization voltage from a nano‐ESI source (Thermo). After collision‐induced dissociation (collision energy 35%, activation Q 0.25, activation time 30 ms) for the top 10 precursor ions, data‐dependent acquisition of centroid MS spectra at 30, 000 resolution and MS/MS spectra were obtained in the LTQ, and the charge determined by the acquisition software is z≥2. Dynamic exclusion of peaks already sequenced was for 20 s with early expiration for two count events with the signal to noise >2. Automatic gating control was set to 150 ms maximum injection time or 10^6^ counts. To identify AEP cleavage sites on synapsin Ⅰ, the SageN Sorcerer SEQUEST 3.5 algorithm was used to search and match MS/MS spectra to a complete semitryptic human proteome database (NCBI reference sequence revision 50, with 66, 652 entries) plus pseudo‐reversed decoys sequences with a 20 p.p.m. mass accuracy threshold (Elias & Gygi, 2007; Xu, Gao, & Ng, 2009). Only *b*‐ and *y*‐ions were considered for scoring (Xcorr) and Xcorr along with ΔCn were dynamically increased for groups of the samples organized by a combination of trypticity (fully or partial) and precursor ion charge state to remove false‐positive hits along with decoys until achieving a false‐discovery rate (FDR) of <5% (<0.25% for proteins identified by more than one peptide). The FDR was estimated by the number of decoy matches (nd) and the total number of assigned matches (nt). FDR =2*nd/nt, assuming mismatches in the original database were the same as in the decoy database. All semitryptic MS/MS spectra for putative AEP‐generated synapsin Ⅰ cleavage sites were manually inspected.

### 4.6 Primary neuronal culture

Primary mouse neurons were cultured as previously described (Zhang et al., 2014). To explore the effect of synapsin Ⅰ C83 fragments on neurons, neurons cultured 7 days *in vitro* (DIV) were infected with AAVs encoding GFP, GFP‐synapsin Ⅰ, GFP‐synapsin Ⅰ C83, respectively. Human synapsin Ⅰ promoter was used to drive neuron‐specific gene expression. The virus was prepared by BrainVTA (Wuhan) Co., Ltd. 7 days later, neurons were fixed in 4% paraformaldehyde and tested by immunofluorescence.

### 4.7 Stereotaxic injection of virus

Unilateral intracranial injection of AAVs was performed stereotactically at coordinates posterior 2.06 mm, lateral 2.35 mm, and ventral 2.35 mm (CA3 region of hippocampus) relative to the bregma in wild‐type and 2‐month‐old tau P301S mice. 200 nl of viral suspension containing 1×10^9^ vector genomes (vg) was injected using a 10‐μl glass Hamilton syringe with a fixed needle.

### 4.8 Generation of antibody that specifically recognizes synapsin Ⅰ C83 fragment (anti‐synapsin Ⅰ C83 antibody)

To generate the anti‐synapsin Ⅰ C83 antibody, the peptide AVKQTTAAAC was used to immunize two rabbits. The rabbits were boosted 4 times with the immunizing peptides with 3‐week intervals between injections. The titers against the immunizing peptide were determined by ELISA. The maximal dilution giving a positive response with the chromogenic substrate for horseradish peroxidase was 1:512,000. The immunoactivity of the antiserum was further confirmed by Western blotting and immunohistochemistry.

### 4.9 Morris water maze test

Five‐month‐old tau P301S mice were trained with extra maze cues as described previously (Zhang et al., 2014). Each subject was tested four times per day for 4 consecutive days, with a 15 min intertrial interval. If the subjects did not find the platform within 60 s, they were manually guided to it, and they had 15 s learning time. The platform was removed on day 5 and the percentage of time spent in the quadrant was measured over 60 s. All trials were analyzed for latency and swim speed using ANY‐Maze software (San Diego Instruments).

### 4.10 Y‐maze test

The Y‐maze test was carried out as described previously (Ma MX et al., 2007). Each arm was 40 cm long, 12 cm high, 3 cm wide at the bottom, and 10 cm wide at the top. The arms converged in an equilateral triangular central area that was 4 cm at its longest axis. The three arms were randomly designated. Start arm: The mouse started to explore when the arms were always open. Novel arm: The arm was blocked during the first trial but open during the second trial. The Y‐maze test consisted of two trials separated by an intertrial interval (ITI) to assess spatial recognition memory. The first trial (training) lasted for 5 min and allowed the mouse to explore only two arms (start arm and another arm) in the maze, with the third arm (novel arm) being blocked. The series of arm entries were recorded visually. After a 2 h ITI (Wang et al., 2007), the second trial (retention) was conducted, during which all three arms were accessible, and novelty vs. familiarity was analyzed by comparing behavior in all three arms. For the second trial, the mouse was placed back in the maze in the same starting arm, with free access to all three arms for 5 min. Recordings were taken and later analyzed using the ANY‐Maze software, and the number of entries and time spent in each arm were analyzed.

### 4.11 Electrophysiology

The brain slides of tau P301S mice were used in electrophysiology experiments. Mice were deeply anesthetized. When all pedal reflexes were abolished, brains were removed and dropped in an ice‐cold oxygenated cutting solution containing the following: 25‐mM D‐glucose, 2.5‐mM KCl, 1.26‐mM NaH_2_PO_4_, 25‐mM NaHCO_3_, 7.2‐mM MgCl_2_, 0.5‐mM CaCl_2_, 3.1‐mM Na‐pyruvate, 11.35‐mM ascorbic acid, and 97‐mM choline chloride. Coronal slices (350‐μm‐thick) containing the dorsal hippocampus were cut at 4°C in the cutting solution using a Leica VT1000S vibratome and then transferred to an incubation chamber filled with oxygenated artificial cerebrospinal fluid (a‐CSF), which contains the following: 118‐mM NaCl, 2.5‐mM KCl, 1‐mM NaH_2_PO_4_, 26‐mM NaHCO_3_, 2‐mM MgCl_2_, 2‐mM CaCl_2_, and 22‐mM glucose in a 35 °C water bath for 30 min and then put in room temperature for 30 min before being recorded. For LTP, brain slices were laid down in a chamber with an 8×8 microelectrode array in the bottom planar (each 50 × 50 μm in size, with an inter‐polar distance of 150 μm) and kept submerged in a‐CSF, with 1–2 ml/min continuing perfusion, with a platinum ring glued by nylon silk. When the noise is stable, electrophysiological signals were acquired using the MED64 System (Alpha MED Sciences, Panasonic). The fEPSPs in CA1 neurons were recorded by stimulating the Schaeffer fibers from CA3. LTP was induced by applying three trains of high‐frequency stimulation (HFS; 100 Hz, 1‐s duration).

For the electrophysiological recording of cultured neurons, neurons were infected with AAV‐synapsin Ⅰ, AAV‐synapsin Ⅰ C83 at DIV7. Seven days later, the neurons cultured in a glass cover were transferred to a chamber perfused with the standard bath solution containing 145‐mM NaCl, 5‐mM KCl, 2‐mM CaCl_2_, 2‐mM MgCl_2_, 10‐mM glucose, and 10 HEPES (pH 7.40, ~310 mOsm). Cultured neurons were recorded with patch pipettes (6–8 MΩ) filled with artificial intracellular fluid (100‐mM CsCH_3_SO_3_, 20‐mM KCl, 10 HEPES, 4‐mM Mg‐ATP, 0.3‐mM Tris‐GTP, 7‐mM Tris2‐Phosphocreatine, 3‐mM QX‐314, pH 7.3, 285–290 mOsm). Neurons were voltage‐clamped at −70 mV with a Multiclamp 700B amplifier, and data were digitized with a Digidata 1550 and analyzed by pClamp 10.0 (Molecular Devices), mEACs were recorded at 32 °C in a bath solution containing 0.5‐μM TTX and 10‐μM bicuculline. Individual events were counted and analyzed with MiniAnalysis program.

### 4.12 FM dye uptake assay

FM dye imaging was performed as described previously (Gaffield & Betz, 2006). Briefly, to load the VS with the FM 4–64 dye, the neurons were incubated in high‐K^+^ Tyrode's solution (90 mM) with 10‐mM FM 4–64 dye for 1–2 min. The cells were switched to normal Tyrode's solution in the presence of FM 4–64 for 15–20 min to recover the nerve terminal. The staining solution was washed out of the imaging chamber with a dye‐free solution. The FM 4–64 fluorescence intensity of boutons was measured by ImageJ software (*n* = 50–70 cells in each group).

### 4.13 FM dye release assay

To load the SVs with the FM 4–64 dye, the neurons were first stimulated with high‐K^+^ Tyrode's solution (90 mM) containing 10‐mM FM 4–64 dye for 1–2 min. Then the cells were switched to normal Tyrode's solution in the presence of FM 4–64 for 15–20 min to allow for complete endocytosis of all released vesicles. The staining solution was washed out of the imaging chamber with a dye‐free solution. The FM 4–64 fluorescence intensity of boutons was considered the basal intensity. Then the cells were stimulated with high‐K^+^ Tyrode's solution (90 mM) without FM 4–64 dye for 3–5 min to induce vesicle releasing, and then washed with normal Tyrode's solution without FM 4–64. The fluorescence intensity was also calculated. The difference between the two fluorescence intensities was considered as FM dye release.

### 4.14 Electron microscopy of synapses

Synaptic density was detected by electron microscopy as described previously (Zhang et al., 2014). After deep anesthesia, mice were perfused transcardially with 2% glutaraldehyde. Hippocampal slices were postfixed in cold 1% OsO_4_ for 1 h. Samples were prepared and examined using standard procedures. Ultrathin sections (90 nm). were stained with uranyl acetate and lead acetate and viewed at 100 kV in a JEOL 200CX electron microscope. Synapses were identified by the presence of SVs and postsynaptic densities.

### 4.15 Golgi staining

Mice were deeply anesthetized, and the brain was removed from the skull as quickly as possible. Rapid Golgi staining was performed using a kit (FD Neuro Technologies, Inc, PK401). Briefly, brain tissue was immersed in silver impregnation solution for 2 weeks in the dark, cryoprotected at 4 °C for 72 h in the dark, and then cut into 100‐μm sections. After sectioning and mounting on gelatin‐coated slides, sections were developed and clarified. To measure the spine density, all clearly evaluable areas of 50~100 μm of secondary dendrites from each imaged neuron were used.

### 4.16 Statistical analysis

All data are presented as mean values ± SEM (standard error of the mean). Statistical analysis was performed using either Student's *t*‐test (two‐group comparisons) or one‐way ANOVA followed by an LSD post hoc test (more than two groups), and *p* values <0.05 were considered significant.

## CONFLICT OF INTEREST

The authors declare no competing financial interests.

## AUTHOR CONTRIBUTION

Z.Z. conceived the project and designed the experiments. L. M. performed most of the experiments. L.Z., H.C., and Z.W. performed electrophysiology experiments. M.X., X.Z., T.Y., and G.C. assisted with immunostaining, Western blotting, and animal experiments. Y.L. and C.L. assisted with data analysis. L.M. wrote the manuscript with input from all authors. K.Y. assisted with data interpretation and critically read the manuscript.

## Supporting information

Fig S1‐S3

## Data Availability

The authors declare that when there is a reasonable request, the authors shall request all the data contained in this study.
