# Supplementary figures and images for: A synapsin Ⅰ cleavage fragment contributes to synaptic dysfunction in Alzheimer's disease
Source: Aging Cell. 2022 Apr 20;21(5):e13619. doi: 10.1111/acel.13619 (PMC9124304; doi:10.1111/acel.13619)

Figure S1

A

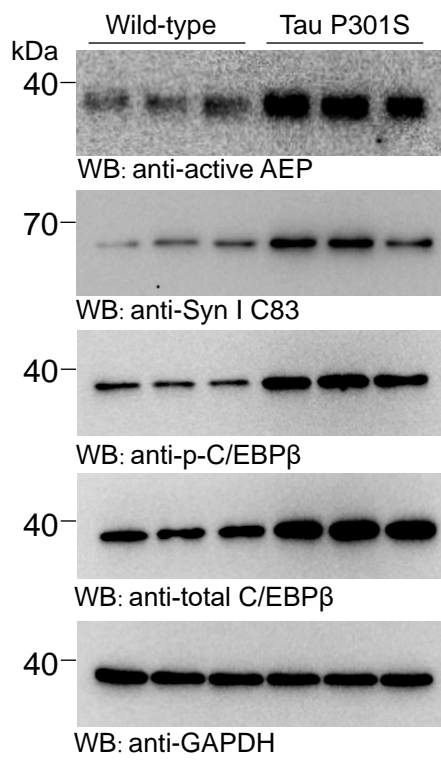

B

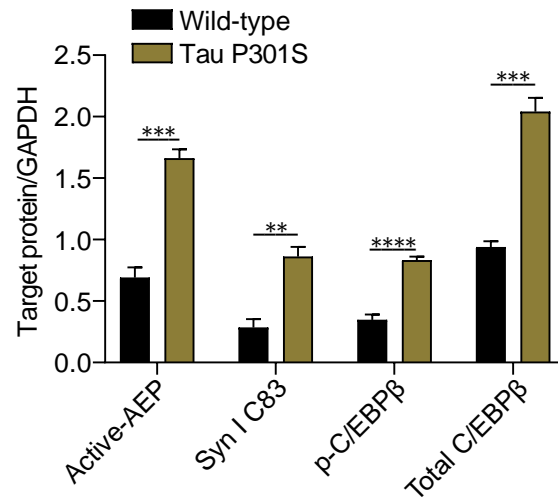

Figure S2

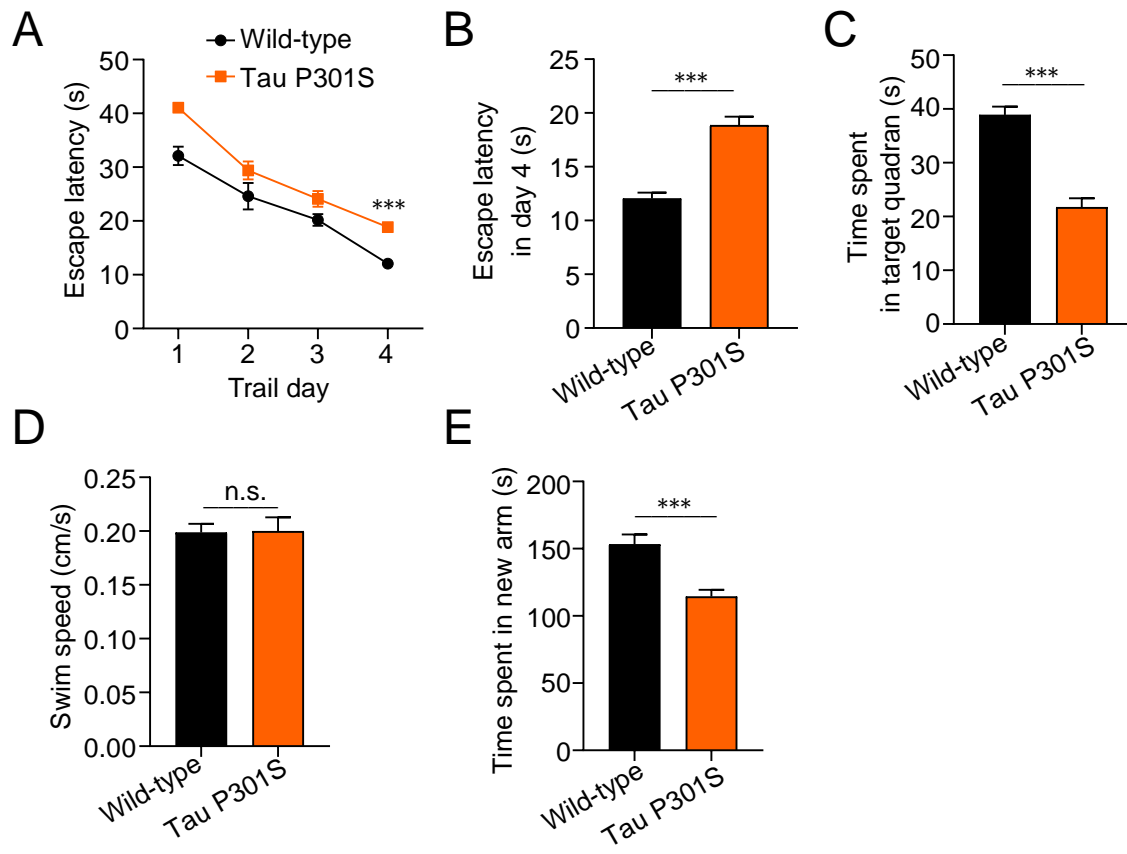

Figure S3

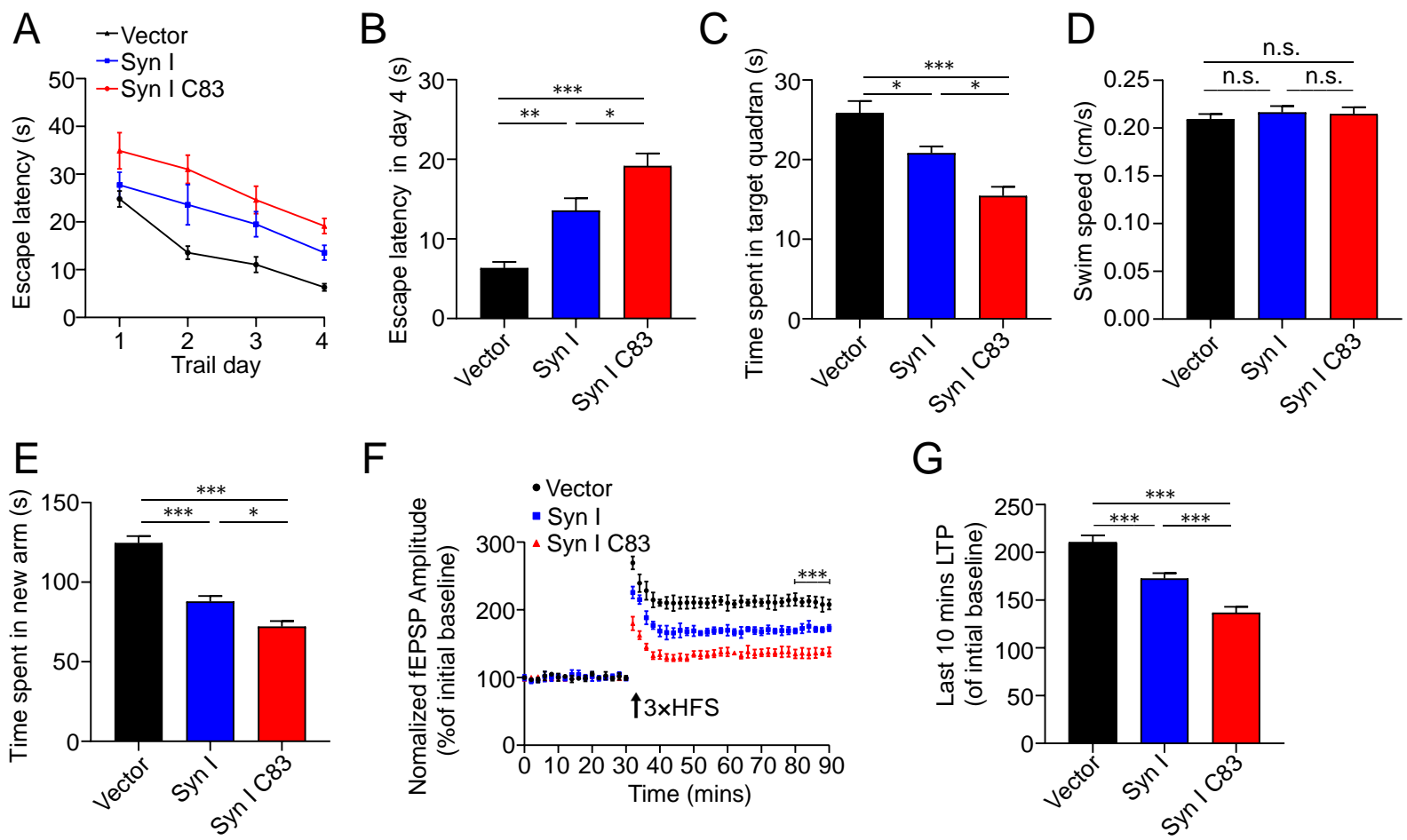

Supplement: Supplementary file 1 — Fig S1‐S3 [file ACEL-21-e13619-s001.pdf]
